# The unexpected finding of a hemangioblastoma on the cerebellum of a patient undergoing treatment with natalizumab for multiple sclerosis

**Published:** 2017-04-04

**Authors:** Yara Dadalti Fragoso, Joseph Bruno Bidin Brooks, Mateus Reghin Neto

**Affiliations:** 1 Neuroimmunology Unit, Department of Neurology, Universidade Metropolitana de Santos, SP, Brazil; 2 Institute of Neurological Sciences, Hospital Beneficencia Portuguesa Sao Paulo, SP, Brazil

**Keywords:** Multiple Sclerosis, Natalizumab, Brain Neoplasms

Natalizumab, a humanized recombinant monoclonal antibody used for the treatment of multiple sclerosis (MS), affects the flow of lymphocytes into the central nervous system (CNS). The drug binds to the alpha-4 chain of the alpha-4-beta-1 integrin (very late activation antigen 4 or VLA-4) and alpha-4-beta-7 integrin. Natalizumab decreases the numbers of CD4+ and CD8+ T lymphocytes, CD19+ B cells and CD138+ plasma cells in the cerebrospinal fluid of patients with MS receiving this therapy.^1^ Thus, at least in theory, this mechanism of action could compromise the immune surveillance within the CNS^2^ and favor the growth of tumors. There have been reports on primary central nervous system lymphoma in patients undergoing treatment with natalizumab,^3^^-^^5^ but the association between these two findings has been deemed unlikely by some.^6^

We want to report a case of hemangioblastoma of the cerebellum in a patient undergoing treatment with natalizumab. The present report was approved by the Ethics Committee at Universidade Metropolitana de Santos and the patient gave consent to its publication, provided confidentiality was guaranteed. Disability is described using the expanded disability scale score (EDSS).^7^

The patient was a woman who is now aged 38 years, with MS diagnosed when she was 28 years old. She was initially prescribed glatiramer acetate, which provided adequate disease control for two years (EDSS: zero). Feeling much better, the patient interrupted her treatment and follow-up for one year, but returned at the age of 31 years presenting acute disease relapse. She was treated with pulses of corticosteroids, but disease control with glatiramer acetate or interferon beta was not achieved. She progressed with relapses and accumulation of disability, presenting tetraparesis, ataxia, severe gait limitations, dysphonia and dysarthria, nystagmus, cognitive dysfunction, and fatigue. Her magnetic resonance imaging (MRI) showed high lesion burden and acute demyelination. The patient started treatment with natalizumab at the age of 34 years and complete control of relapses and lesions, as seen on MRI, was achieved without further accumulation of disability (EDSS: 5.5) over the course of 44 monthly infusions of the drug, and did not show any new neurological signs or symptoms. A cystic formation was then detected in the cerebellum on her yearly routine MRI (Figure 1). Total surgical resection 90 days ago (April 2016) confirmed the diagnosis of hemangioblastoma (Figure 1). 

**Figure 1:**
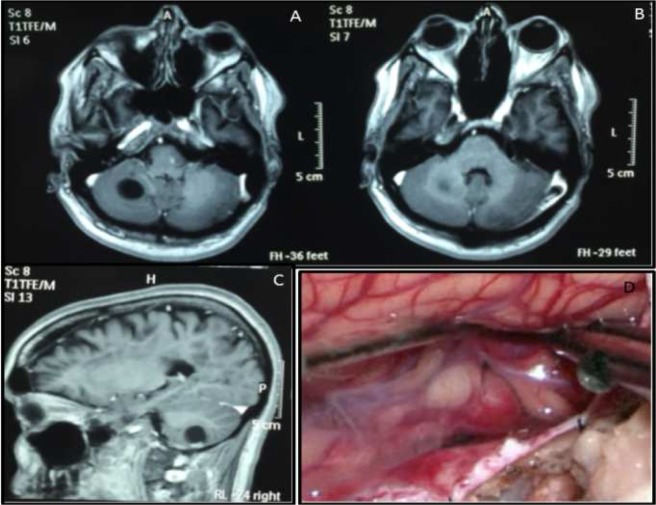
Magnetic resonance imaging (MRI) of a cystic formation on the cerebellum (A, B and C); detail of surgical resection of the tumor (D), later confirmed to be an hemangioblastoma

The patient has been stable since surgery, without further disability, and natalizumab has been withdrawn. There is a previous report of a patient with MS presenting von Hippel-Lindau syndrome.^8^ This condition leading to cystic tumors was investigated and excluded in the patient reported here.^9^

Although no causal relationship can be established between use of natalizumab and this finding of hemangioblastoma, these are rare tumors. The finding may have been purely coincidental, but the purpose of the present report was to contribute further on the discussion of a potential association between insufficient immune surveillance and brain tumors.

Disclosure: The authors have no conflicts of interest to declare. Pharmacovigilance at Biogen Idec has been informed of the adverse event.

## Notes:

**How to cite this article:** Fragoso YD, Bidin Brooks JB, Neto MR. The unexpected finding of a hemangioblastoma on the cerebellum of a patient undergoing treatment with natalizumab for multiple sclerosis. Iran J Neurol 2017; 16(2): 96-7.
